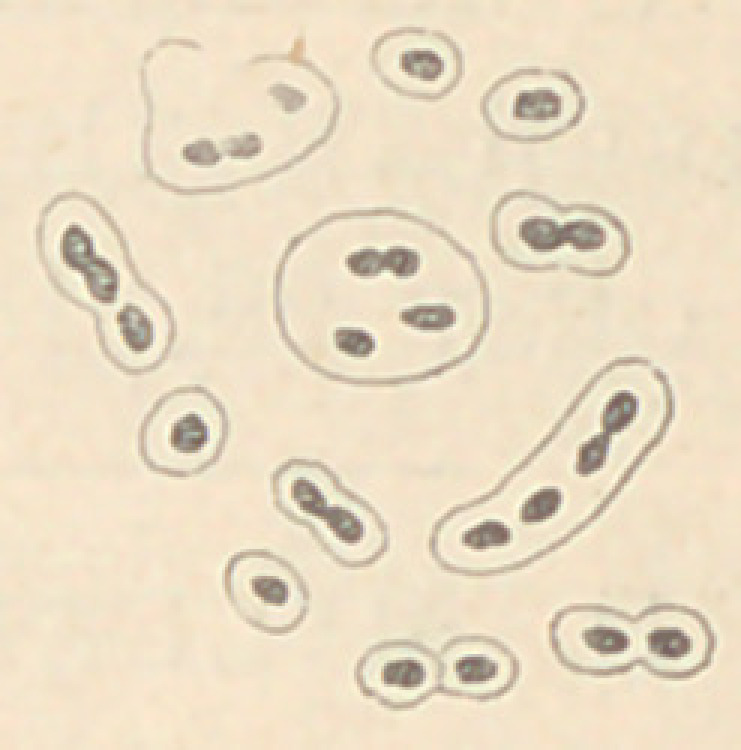# A Case in Practice

**Published:** 1885-01

**Authors:** W. D. Miller

**Affiliations:** Berlin


### A CASE IN PRACTICE.

PRESENTED BEFORE THE AMERICAN DENTAL SOCIETY OF EUROPE, AUGUST 28, 1884.

BY W. D. MILLER, BERLIN.

The following case may serve to demonstrate the pathogenic nature of certain micro-organisms which are occasionally, to say the least, to be met with in the human mouth, and the consequent possibility of the infection of either patient or operator through wounds incurred during the performance of dental operations.

Frau X., somewhat anæmic, otherwise in good health, called upon me some weeks ago, complaining about often having a taste of old Limburgh cheese in her mouth. I immediately examined the dental arch without finding a trace of suppuration anywhere, and but very slight decay. Turning my attention to the throat, I at once discovered the cause of the complaint, the very much enlarged tonsils being almost concealed by a thick, yellowish-white coating, which was found to consist of masses of many different fungi, cocci and diplococci predominating, some of the latter appearing to be surrounded by a delicate gelatinous sheath. The coating upon the surface of the glands was removed without much difficulty, partly mechanically, partly by means of antiseptic washes. It was found, however, that the fungi had penetrated the fissures between the folds of the tonsils in some places to the depth of one-fourth, or one-half an inch, where they were entirely secure against the action of the most powerful antiseptics superficially applied.

After a great many unsuccessful attempts, by myself as well as by the family physician of the patient, I accomplished a partial success by dipping a delicately curved probe into a ninety per cent.

solution of carbolic acid, and then working it between the folds of the glands wherever nests of fungi had been established.

A complete eradication has, however, up to the present day, not been accomplished.

When this case was first observed, a small quantity of the saliva was brought into about fifty c. c. of sterilized calves' broth, and allowed to stand for four or five hours at thirty-seven degrees C. At the end of this time one c. c. of the solution was injected into the lung of a full grown rabbit. Death occurred in thirty hours, the blood of the animal being densely crowded with cocci and diplococci, surrounded for the most part by a wide sheath, best seen when stained with a solution of gentian-violet in aniline water.

The subjoined cut will give an idea of the appearance of this fungi under the microscope.

## Figures and Tables

**Figure f1:**